# Machine learning in hypertension: from diagnosis to personalised medicine

**DOI:** 10.1016/j.ebiom.2023.104658

**Published:** 2023-06-14

**Authors:** eBioMedicine

Hypertension, also known as high blood pressure, is defined as having a sustained blood pressure of 140/90 mm Hg or higher and is a major risk factor for cardiovascular disease, renal complications, and premature death worldwide. World Hypertension Day occurs every year on May 17 to increase awareness and educate the public about the condition. Estimates suggest that 1.28 billion adults aged 30–79 years worldwide are living with hypertension, of whom 46% are unaware that they have the condition and only 42% are diagnosed and treated.

Patient care has evolved substantially in the past few decades, with several guidelines and treatment goals published by different organisations (eg, the American College of Cardiology and American Heart Association, the International Society of Hypertension, and the National Institute for Health and Care Excellence). However, many aspects associated with hypertension are still poorly understood, such as its underlying causes and contributing factors, and several shortfalls persist, including treatment optimisation and adequacy of blood pressure control.

With the increased digitalisation of health care, large amounts of generated data have become available to clinicians and researchers, and machine learning could be used to gain valuable insights into complex medical conditions. For hypertension, the uses and opportunities for machine learning are broad and could ultimately support informed decision making by offering insights into hypertension prevalence and risk, diagnosis and severity, risks of subsequent complications, and patient management and treatment.

Accurate disease diagnosis is a crucial step in patient care as it substantially improves the likelihood of positive health outcomes. Traditional studies on hypertension have often assessed only single mechanistic pathways via conventional approaches involving statistical tools. However, these approaches have a reduced capacity to capture the multitude and complexity of factors affecting blood pressure (eg, molecular and physiological), thus limiting the understanding of the condition. The use of machine learning algorithms can facilitate the integration of multi-omics data to aid the discovery of biomarkers for diagnosis. In *eBioMedicine* (2022), Louca and colleagues used an extreme gradient boosting (XGBoost) algorithm with 5-fold cross-validation to understand the multifactorial contributors to blood pressure. The authors analysed multimodal data, including genetic, metabolic, laboratory, and demographic features, to predict systolic and diastolic blood pressure measurements in two ethnically diverse populations (ie, the TwinsUK and Qatari biobank cohorts). The model explained a large proportion of the variance in systolic blood pressure in participants from the TwinsUK cohort (39.2%, 95% CI 34.7–43.7) and the Qatari biobank cohort (45.2%, 31.8–58.6). Traditional risk factors (ie, age and BMI), metabolites (ie, dihomo-linolenate and cis-4-decenoyl carnitine), biochemical measures (ie, lactate, chloride, urate, and creatinine), and dietary intakes of total, trans, and saturated fat were the most predictive features of blood pressure, whereas genetic factors (ie, single nucleotide polymorphisms) were not. In another study in *eBioMedicine* (2022), Reel and colleagues showed that the integration of multi-omics via machine learning had higher discriminatory power than single-omics (ie, mono-omics) data analysis, and correctly classified different forms of endocrine hypertension with high sensitivity and specificity, providing potential diagnostic biomarker combinations for diagnosing secondary hypertension (ie, hypertension with a known cause) subtypes.

Machine learning can also substantially contribute to the prognosis of complex conditions because of its ability to process large amounts of complex data. The identification of patterns and prediction of outcomes could inform prognoses and guide disease management or therapy (eg, on the basis of risk stratification). In *Hypertension* (2020), Wu and colleagues combined recursive feature elimination and XGBoost to build a model predicting clinical outcomes in young patients with hypertension. The performance of the machine learning method (concordance statistic 0.757, 95% CI 0.660–0.854) was similar to the prognostic efficacy of the Cox regression model (0.723, 0.636–0.810; p = 0.500) and higher than the prognostic efficacy of the recalibrated Framingham risk score model (0.529, 0.403–0.655; p = 0.006). Although the machine learning method used variables that might not be as regularly available as those needed to calculate the other models (eg, mean nocturnal arterial oxygen saturation, blood pressure measurements of all limbs, and urea), some of these variables might represent new predictors of hypertension. In *Diagnostics* (2019), Chang and colleagues applied recursive feature elimination with cross-validation to identify which input variables provided better predictive power to train four different classification algorithms for hypertension outcomes based on medical data. Of the four classifiers, the XGBoost algorithm produced the best results, with a prediction performance of 94.36% and an area under the receiver operating characteristic curve of 0.927.

Machine learning could also aid the prediction of optimal hypertension treatments and facilitate a personalised medicine approach. Researchers and clinicians could identify how treatment might vary between patients based on individual characteristics, with the potential of increasing treatment effectiveness and improving population health outcomes. Ye and colleagues in the *International Journal of Medical Informatics* (2020) and Hu and colleagues in *BMC Medical Informatics and Decision Making* (2023) applied different machine learning tools to identify optimal treatment pathways using electronic health records data. Their findings suggest the utility of predictive models as robust decision-making tools to form risk-adapted, personalised treatment strategies. Oikonomou and colleagues in *The Lancet Digital Health* (2022) and Inoue and colleagues in the *International Journal of Epidemiology* (2023) used data from two randomised clinical trials—the SPRINT and ACCORD BP trials—investigating the benefits of intensive systolic blood pressure control versus standard treatment. Oikonomou and colleagues developed a machine-learning-based tool that identified individualised treatment effects using participant-level data for individuals with and without diabetes. Inoue and colleagues used a machine learning technique known as causal forest to identify patients who would benefit the most from blood pressure management. The machine-learning-based high-benefit approach (ie, treatment of individuals with high estimated benefit) outperformed the high-risk approach (ie, treatment of individuals considered at high risk), providing a larger treatment effect. The high-benefit approach might improve treatment effectiveness and population health outcomes without additional resources by identifying people with a high estimated individualised treatment effect, which is a function of individual observable characteristics, regardless of whether they meet the diagnostic criteria for hypertension.

In 2023, an EU-funded project, HYPERMARKER, was launched to develop and test tools that include machine learning approaches to leverage the use of pharmacometabolomics and identify the most suitable antihypertensive treatment for patients with hypertension. Data will be obtained from well phenotyped cohorts from 11 European countries and the prediction models will be clinically validated through a randomised controlled trial across four sites in Europe. The outcome will be a clinical decision support tool that will allow clinicians to make an informed choice regarding medication for individual patients (ie, targeted treatments), thus potentially improving their outcomes.

There are several challenges associated with machine learning tools, such as the need for external validation and transparency for patients, which are strongly encouraged at *eBioMedicine*. Other issues of clinical applicability might be more difficult to avoid or mitigate and include poor interpretability and potential biases associated with inappropriate design, unrepresentative data, and human biases. Despite these challenges, machine learning has great promise for the future of health care. The clinical implementation of machine learning is evolving and will be pivotal in increasing our comprehension of diseases, such as hypertension, by unravelling the underlying complexities associated with early diagnosis, prognosis, and discovery of potential therapies. As a translational publishing platform, *eBioMedicine* welcomes research on the discovery and validation of biomarkers and mechanisms of hypertension via machine learning tools. Open access to data, code sharing, and external validation are essential for studies to be considered at *eBioMedicine*.

